# Knowledge and practices of toxoplasmosis among healthcare workers at two large referral hospitals in Zambia: Implications on the One Health Approach

**DOI:** 10.1371/journal.pgph.0002235

**Published:** 2023-08-15

**Authors:** Victor Daka, Moses Mukosha, Scott K. Matafwali, Steward Mudenda, Andrew M. Phiri

**Affiliations:** 1 Department of Clinical Studies, School of Veterinary Medicine, University of Zambia, Lusaka, Zambia; 2 Public Health Department, School of Medicine, Copperbelt University, Ndola, Zambia; 3 Africa Center for Infectious Diseases of Humans and Animals, University of Zambia, Lusaka, Zambia; 4 Department of Pharmacy, School of Health Sciences, University of Zambia, Lusaka, Zambia; 5 Clinical Research Department, Faculty of Infectious and Tropical Diseases, London School of Hygiene &Tropical Medicine, London, United Kingdom; Federal Fluminense University: Universidade Federal Fluminense, BRAZIL

## Abstract

**Introduction:**

Assessing the knowledge and practices of healthcare workers regarding *Toxoplasma gondii* infection, diagnosis, treatment, and control is crucial for developing an effective management strategy.

**Methods:**

A cross-sectional study was conducted among 175 healthcare workers at Ndola Teaching Hospital and Namwala District Hospital in Zambia from September 2021 to April 2022.

**Results:**

More than half (57.1%) of the respondents were males. Overall, 46(26.3%) and 68 (38.9%) respondents reported good knowledge and practices, respectively. Respondents with a higher number of years of experience (AOR = 0.86, 95% CI: 0.77–0.97), who were nurses than clinicians (AOR = 0.17, 95% CI: 0.007–0.41) and working at Ndola teaching hospital than Namwala hospital (AOR = 0.34, 95% CI:0.13–0.89) were less likely to have good knowledge. Respondents with a degree qualification than a diploma (AOR = 3.04, 95% CI: 1.09–8.47) were more likely to have good knowledge. Respondents from Ndola teaching hospital than Namwala hospital (AOR = 0.40, 95% CI: 0.17–0.92) were less likely to have good practices.

**Conclusion:**

Our study revealed that healthcare workers had low levels of knowledge and poor practices, which could have negative implications for the management of toxoplasmosis. To improve their knowledge and practices, continuous medical education in *Toxoplasma* related aspects is recommended for in-service healthcare workers.

## Introduction

Toxoplasmosis, caused by the obligate intracellular Apicomplexan parasite *Toxoplasma gondii* (*T*. *gondii*), is distributed worldwide and can infect both humans and animals [1–3]. It is estimated that a third of the world’s population is infected with *T*. *gondii*, making it one of the most successful parasites and a global health hazard [4, 5].

Although it is relatively benign in individuals with a competent immune system, it may cause significant morbidity in the immunocompromised, where it can reactivate latent infection leading to cerebral complications [6, 7]. An acute infection with *Toxoplasma gondii* during pregnancy can lead to severe congenital complications for the fetus, with the highest risk of complications in the first trimester compared to the third trimester. However, the risk of infection increases as the pregnancy progresses [7, 8]. Congenital complications include seizures, deafness, blindness, mental retardation and stillbirth [9, 10]. The prevention of congenital and cerebral toxoplasmosis relies heavily on healthcare worker’s knowledge [4, 7, 11]. Despite its global importance, toxoplasmosis has been reported infrequently in humans in Zambia, with evidence of endemicity in different settings. Studies by Zumla and others reported a seroprevalence of 4% and 11% in HIV-positive and HIV-negative individuals, respectively [12]. More recently, a study conducted in Ndola, a Northern part of Zambia, reported a seroprevalence of 12.4% in HIV-positive, 9.5% in HIV-negative individuals and 9.2% in women of childbearing age [13]. A study in pregnant women found a *T*. *gondii* IgG seroprevalence of 5.87% at a large referral hospital in Lusaka [14]. Currently, toxoplasmosis is not part of the healthcare benefits package in Zambia and has not been included in the national strategic plan for neglected tropical diseases.

Knowledge of toxoplasmosis has been known to improve the prevention and control of the disease, driven by inherent good practices towards the disease [15, 16]. There are variable reports on knowledge and practices of toxoplasmosis among healthcare workers. Low levels of knowledge have been reported in Brazil among physicians and nurses [17, 18]. In Mexico, low knowledge levels were reported among medical laboratory professionals. This was also consistent with poor practices, with 84.4% of the respondents reporting not having performed a laboratory test to detect of *T*. *gondii* [4]. In contrast, good knowledge has been reported in Nigeria among medical doctors and in Morocco among healthcare workers [7, 11].

Because the transmission of T. gondii is zoonotic and environmental in nature, a One Health Approach is imperative [19]. This must be driven by evidence-based measures augmented by robust scientific data and a strong knowledge base among caregivers and the general population as their close interation with pateints also facilitates knowledge transfer regarding prevention and control [20]. Despite the limited data on toxoplasmosis in Zambia, the knowledge and practices of Healthcare Workers (HCWs) regarding the infection are crucial for effective management strategies as well as triggering interest in more research. This study investigated knowledge and practices regarding toxoplasmosis in Namwala and Ndola districts of Zambia.

## Materials and methods

### Study design

This was a cross-sectional study among health workers at Namwala District Hospital and Ndola Teaching Hospital between September 2021 and April 2022.

### Study setting

Namwala District Hospital is located in the rural district of Namwala in the Southern province of Zambia. The hospital has a bed capacity of 52 beds with a catchment population of predominantly farmers. The district covers an estimated total area of 10,000 km^2^ and lies between latitudes 15° and 17° South of the equator and longitude 25° and 27° [21]. There is a very thin animal and wildlife interface with many shared ecological habitats, such as water drinking points which increases the possibility of zoonotic disease transmission such as toxoplasmosis [22]. The hospital is the main referral facility, supporting other rural clinics and health centres in the district.

Ndola Teaching Hospital is located in the Copperbelt Province of Zambia, an urban area of northern Zambia. It is a referral hospital with a bed capacity of 851 and supports districts in Copperbelt, Luapula and North-Western Provinces of Zambia. Its catchment population is mainly composed of formally employed individuals and attends to referrals of different socio-economic statuses from the provinces it covers [23, 24].

**Fig 1:**
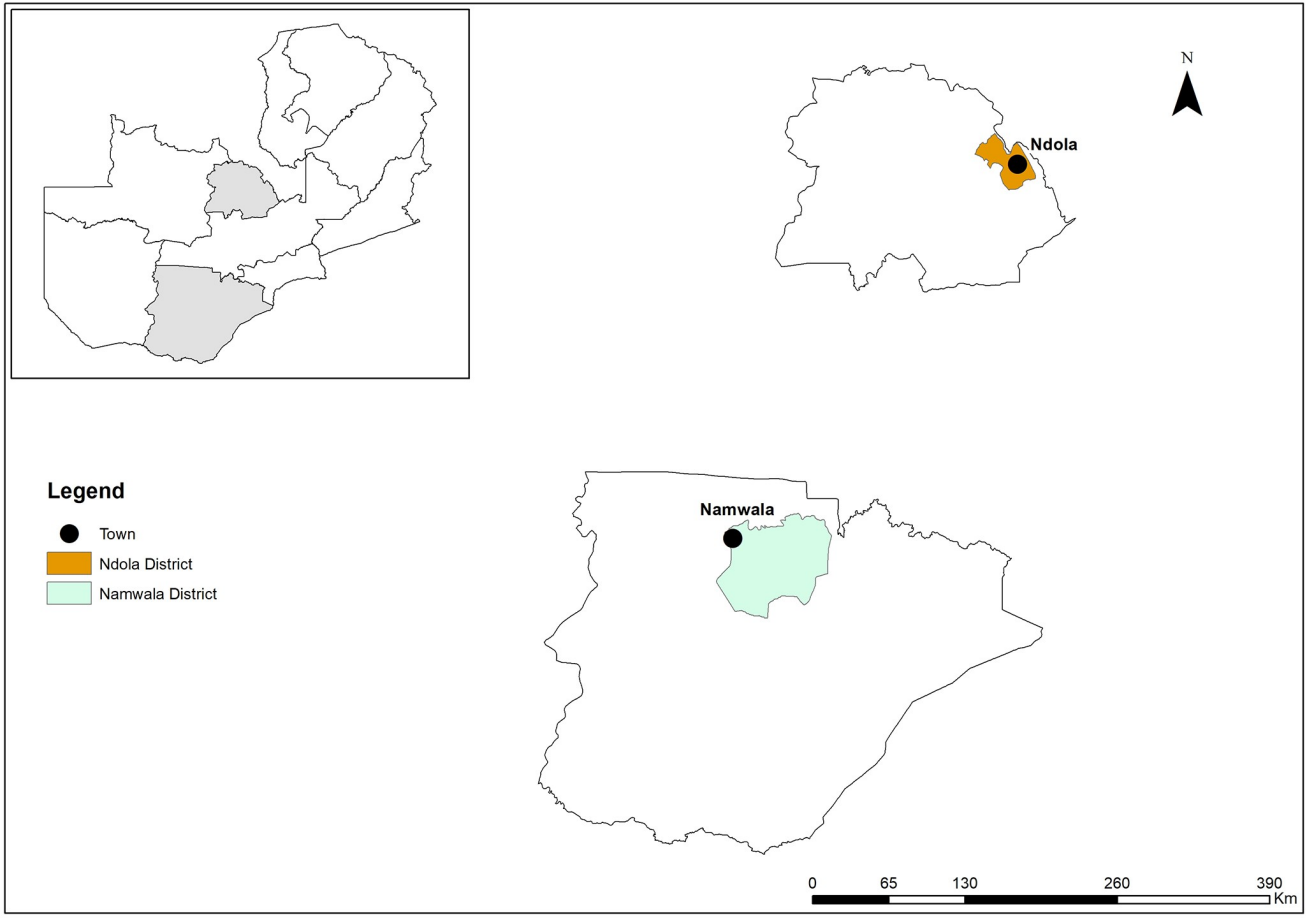
Map of Zambia showing Namwala and Ndola districts of Zambia. [Map created by ArcGIS software (Environmental Systems Research Institute (ESRI), Redlands, California) and source of the basemap shapefile was from https://data.amerigeoss.org/fi/dataset/zambia-administrative-boundaries-level-1-provinces-and-level-2-districts-with-census-2010-popul].

The hospital selection was performed conveniently with a focus on obtaining data from HCWs in both urban (Ndola) and rural (Namwala) settings (Fig 1).

### Study population

Participants were purposefully selected considering their regular interactions with high-risk groups, including pregnant women and patients on anti-retroviral therapy. These included clinicians, biomedical personnel, pharmacy personnel and nurses. Radiographers, environmental health personnel and community health assistants involved in the management of toxoplasma risk groups were classified as ‘Other’. A total of 75 and 240 HCWs were identified from Namwala District Hospital and Ndola Teaching Hospital, respectively. A total enumberation selection of participants was carried out with all HCWs present during the period of the study being approached to be part of the study in their designated areas of work until the desired sample size required was achieved.

### Sample size determination

Open Epi Software was used to calculate the minimum sample size from the 315 HCWs in the two hospitals [25]. We employed a conservative estimate of 50% knowledge of toxoplasmosis due to the absence of information on knowledge of toxoplasmosis in Zambia. Factoring in an acceptable margin error of 5% at 95% confidence level and correcting for our finite population of 315 HCWs, we estimated a minimum sample size of 174 participants. A total of 240 health workers were provided with the questionnaire, and 175 responded giving a response rate of 73%. Participants were enrolled continuously at both sites until the desired proportional representation based on population size was reached for each site. A total of 41 and 134 participants were enrolled in the study from Namwala and Ndola districts, respectively.

### Data collection

Data were collected by the REDCap application installed on a mobile tablet [26]. The questionnaire was administered by a trained data collector. We adapted the questionnaire from previous studies and formulated the questions to our context [11]. Informed consent was administered to all eligible HCWs present during the period of the study. All those that consented by signing the consent form were then assigned a study identification number, and then the questionnaire was administered.

We performed content validation by administering the questionnaire to 30 HCWs at Arthur Davison Children’s hospital in Ndola, Zambia. The results from this pretest were censored from the final analysis, and feedback from the participants was used to optimise the content of the questionnaire for coherence and logic. The questionnaire had four main components: Section A was on facility details and had four questions; section B was on demographic and background characteristics and had 13 questions; section C was on knowledge and had 30 questions, and section D was on risk factors and had nine questions (S1 File).

### Data management and statistical analysis

Data were entered and managed using the REDCap mobile and web-based applications. Further data cleaning was done in Microsoft Excel@ and then exported to STATA version 17/BE (Stata Corp., College Station, Texas, USA) for statistical analysis employing complete case analysis according to methods described elsewhere [27]. We used the Cronbach’s alpha to check for the internal reliability of the collected data and obtained an alpha value of 0.724, indicating adequate reliability [28]. We did not stratify the sample among the HCWs because we assumed that the measured variables were equal. For each scale (knowledge and practice scales), the item scores were summed to create a score. The percentage scores were obtained by dividing the actual score by the total possible score and multiplying by 100. Using the bloom’s cut point of 60%, the percentage scores of knowledge and practice were categorised as good (above 60%) and poor (60% or below) [29]. Continuous variables (age, years of experience, knowledge and practice scores) were summarised using means and standard deviation (SD) or median, interquartile range (IQR) as appropriate. Categorical variables (HCW’s speciality, health facility, marital status, sex and highest qualification attained) were summarised using frequencies and percentages.

Separate logistic regression models were fitted with “knowledge” and “practice” as the outcome variable and one of the explanatory variables at a time in order to see any evidence of an association between the variable and good knowledge or practice. Then two separate multivariable logistic regression models were fitted with the HCW’s speciality and any additional variables that were significant at 20% in the single models. Variables that were not significant at 20% level in the multivariable models were removed one by one, starting with the ones with the highest percentage. To avoid inflating type I error rate; continuous variables were not categorised in the regression models. Finally, interactions between HCWs’ speciality and the confounding variables remaining in the final knowledge and practice models were assessed.

### Ethical considerations

Ethical approval to conduct this study was granted by the Tropical Diseases Research Centre Ethics Committee (Institutional Review Board Number 00002911), and authority to conduct the study was obtained from the National Health Research Authority. Permission to conduct the study at the facilities was obtained from the Provincial Health Directors. All questionnaires were anonymised and data were restricted to the investigators.

### Inclusivity in global research

Additional information regarding the ethical, cultural, and scientific considerations specific to inclusivity in global research is included in the S3 File.

## Results

Overall, 175 HCWs were enrolled, of whom 100 (57.1%) were males. The largest proportion, 85 (48.6%), were nurses, and the least 6 (3.4%) were other minor professions. The majority, 134 (76.6%) were from Ndola teaching hospital. A total of 122 (68.7%) attained a diploma as the highest qualification. Approximately half, 89(50.9%), were not married. The median age and years of practice was 29 years (interquartile range [IQR], 27–34) and 3 years (IQR, 2–7), respectively.

The overall respondents’ mean knowledge and practice scores were 43.1% ± 23.1 and 55.3% ± 16.7%, respectively (Table 1).

**Table 1:** Socio-demographic characteristics of participants by knowledge and practice of toxoplasmosis preventions in Zambia (N = 175).

Variable	Level	Total population, n (%)	Knowledge, n (%)		Practice, n (%)	
			Poor	Good	Poor	Good
Sex	Female	100(57.1)	79(61.2)	21(45.7)	69(64.5)	31(45.6)
	Male	75(42.9)	50(38.8)	25(54.4)	38(35.5)	37(54.4)
Facility^a^	Namwala	41(23.4)	27(20.9)	14(30.4)	16(15.0)	25(36.8)
	Ndola	134(76.6)	102(79.1)	32(69.6)	91(85.1)	43(63.2)
Marital status	Unmarried	89(50.9)	68(52.7)	21(45.7)	57(53.3)	32(47.1)
	Married	86(49.1)	61(47.3)	25(54.4)	50(46.7)	36(52.9)
Highest qualification attained	Diploma	122(69.7)	99(76.7)	23(50.0)	71(66.4)	51(75.0)
	Degree	53(30.3)	30(23.3)	23(50.0)	36(33.6)	17(25.0)
Years of experience (IQR)		3(2–7)	4(2–7)	2(2–5)	3(2–7)	5(3–7)
Age, median (IQR)		29(27–34)	29(27–35)	29(27–32)	29(27–33)	29.5(27.5–33.5)
Overall score % mean (SD)			43.1(23.1)		55.3(16.7)	

Key: IQR-interquartile range, SD-standard deviation,

^a^Ndola Teaching Hospital and Namwala district hospitals

Table 2 shows evidence of an association between HCW’s speciality, knowledge *(p<0*.*001)* and practice (p = 0.004). Overall, 46(26.3%) of respondents reported good knowledge while 68(38.9%) reported good practices. Of the HCWs who reported good knowledge, the largest proportion 18 (39.1%) were clinicians, followed by biomedical scientists 16 (26.1%), and the least were pharmacy personnel 5 (10.9%). On the other hand, the highest proportion, 24(35.3%), for good practices was reported among the nurses and the least among other minor professions 3(4.4%).

**Table 2:** Healthcare worker’s speciality according to knowledge and practice scores in Zambia.

Healthcare worker	Total population n (%)	Seen or tested a patient for toxoplasmosis	Knowledge		p-value	Practice		p-value
		n = 40(%)	Poor, n = 129(%)	Good, n = 46(%)		Poor, n = 107 (%)	Good, n = 68 (%)	
Clinician	38(21.7)	17(42.5)	20(15.5)	18(39.1)	<0.001	25(23.4)	13(19.1)	0.004
Biomedical	34(19.4)	10(25)	22(17.1)	12(26.1)		12(11.2)	22(32.4)	
Nurse	85(48.6)	12(30)	74(57.4)	11(23.9)		61(57.0)	24(35.3)	
Pharmacy	12(6.9)	0(0)	7(5.43)	5(10.9)		6(5.6)	6(8.8)	
Other	6(3.4)	1(2.5)	6(4.7)	-		3(3(2.8))	3(4.4)	

The logistic regression model that looked at one variable at a time (Table 3) found that the HCW’s speciality, health facility, highest qualification attained, and years of experience were associated with good knowledge. On the other hand, good practices were associated with HCW’s speciality, health facility, and sex of the respondent.

**Table 3:** Association between healthcare worker’s speciality, knowledge and practice, adjusting for potential confounding variables in Zambia.

Variable	Knowledge		Practice	
	UOR [95% CI]	AOR [95% CI]	UOR [95% CI]	AOR [95% CI]
Health worker				
Clinician	Ref	Ref	Ref	Ref
Biomedical	0.61[0.23, 1.56]	0.81[0.24–2.79]	3.53[1.33–9.31]*	2.59[0.93–7.29]
Nurse	0.17[0.07, 0.41]**	0.21[0.05–0.81]*	0.76[0.33–1.72]	0.74[0.26–2.10]
Pharmacy	0.79[0.21, 2.95]	0.87[0.19–4.06]	1.92[0.52–7.16]	1.32[0.32–5.41]
Other^a^			1.92[0.34–10.90]	1.06[0.17–6.68]
Sex				
Female	Ref		Ref	Ref
Male	1.88[0.95, 3.71]		2.18[1.17–4.03]*	1.17[0.49–2.80]
Facility				
Namwala	Ref	Ref	Ref	Ref
Ndola	0.60[0.28, 1.29]	0.34[0.13–0.89]*	0.30[0.15–0.62]**	0.40[0.17–0.92]*
Marital status				
Unmarried	Ref		Ref	
Married	1.33[0.68, 2.61]		1.28[0.70–2.36]	
Highest qualification				
Diploma	Ref	Ref	Ref	
Degree	3.30[1.62, 6.70]*	3.04[1.09–8.47]*	0.66[0.33–1.30]	
Years of experience	0.87[0.78, 0.96]	0.86[0.77–0.97]*	1.01[0.95–1.09]	
Age (years)	0.95[0.88, 1.01]		1.00[0.95–1.06]	

**p<0.01,

*p<0.05,

UOR-unadjusted odds ratio, AOR-adjusted odds ratio, 95% CI-95% confidence interval,

^a^other professions i.e., environmental officers, community health assistants and radiographers

When controlling for modifying variables which were statistically significant at the 20% level in the univariable model (HCW speciality, health facility, highest qualification attained, years of experience, age of respondents), the multivariable logistic regression showed that independent factors associated with good knowledge were HCW specialty, health facility, highest qualification attained and years of experience. Respondents with higher years of experience (AOR = 0.86 for a one year increase in years of practice; 95% CI: 0.77–0.97), who were nurses than clinicians (AOR = 0.17, 95% CI: 0.007–0.41) and working at Ndola teaching hospital than Namwala hospital (AOR = 0.34, 95% CI:0.13–0.89) were less likely to have good knowledge. Conversely, respondents with a degree than a diploma (AOR = 3.04, 95% CI: 1.09–8.47) were more likely to have good knowledge (Table 3).

The multivariable logistic regression with “practice” as an outcome, adjusted for the sex of respondents, HCW’s speciality and health facility based on 20% cut of level from the single models. The final model showed that health facility was independently associated with good practice. Respondents from Ndola teaching hospital than Namwala hospital (AOR = 0.40, 95% CI: 0.17–0.92) were less likely to have good practices (Table 3).

## Discussion

To our knowledge, this is the first study investigating the knowledge and practices of toxoplasmosis in Zambia, forming the basis for developing evidence-based management strategies. The current study reported low levels of knowledge regarding toxoplasmosis aetiology, diagnosis and treatment among respondents. This has negative implications on the role and contribution of healthcare workers who are key contributors to the one health approach of tackling toxoplasmosis [11, 20]. The results of the present study are consistent with similar studies that reported low knowledge levels among HCWs in other countries such as Brazil [17, 18], Ethiopia [30], Tanzania [31] and among medical students in Saudi Arabia [32]. In contrast, higher knowledge levels were seen among HCWs in Nigeria and Morocco [7, 11]. This disparity in knowledge levels could be attributed to differences in target respondents in the studies. Previous studies primarily focused on determining awareness of specific aspects of toxoplasmosis, rather than the level of knowledge.

Our results also show low adherence to good practices among HCWs regarding toxoplasmosis. This could be attributed to the low knowledge. The lack of knowledge and poor practices among HCWs is concerning. They are expected to be a source of knowledge for patients which would lead to positive behaviours for the prevention of diseases such as toxoplasmosis [33]. Our assertion, however, could not be tested as the design of the present study could not investigate cause as in a clinical trial. The present study reports a higher proportion of clinicians having seen a case of toxoplasmosis compared to other categories of staff. However, the overall percentage of staff that had seen or tested for toxoplasmosis was low. This is consistent with other findings [4, 11].

Clinicians had a higher level of knowledge about toxoplasmosis compared to other categories of staff. This is likely due to being the first contacts during case management of patients thereby increasing the likelihood of encountering *Toxoplasma* cases. This is an agreement with a previous study done by Efunshile and others [11]. Additionally, our findings indicate that nurses had lower knowledge levels than clinicians, a finding that is consistent with other studies [11]. This could possibly be due to an absence of information on toxoplasmosis in the formal training of nurses.

Regarding practice, nurses were found to have better practice compared to other professionals. Nurses could be inadvertently exhibiting good practices as they perform their routine work as they are more engaged and aware of the infection control practices in the hospital environment. A previous study on practices among clinical laboratory professionals reported poor practices regarding toxoplasmosis testing, consistent with our findings [4].

In the present study, we reported that staff at Namwala District Hospital had a higher level of knowledge about toxoplasmosis compared to those at Ndola Teaching Hospital. This is in contrast to the findings of Mahfouz and others, who reported that urban populations had higher knowledge levels than rural populations [32]. We postulate that this discrepancy may be due to the smaller size of Namwala District Hospital, which allows for more opportunities for staff to receive training and continuing medical education. In contrast, the larger workforce at Ndola Teaching Hospital may have fewer opportunities for staff to attend scheduled training offered by the Zambian Ministry of Health.

In this study, we found that staff with less working experience had a higher knowledge of toxoplasmosis than those with more experience. This finding is consistent with that from another study in Brazil, where health professionals with more than 10 years of experience had lower knowledge levels than those with 10 or less years [18]. This contradicts the commonly held belief that more experience leads to more significant knowledge acquisition over time [11, 34]. We suggest that this unexpected outcome may be due to a lack of effective continuing medical education programs for staff, which causes the knowledge obtained from formal education to deteriorate over time.

The present study may be limited in that only participants from two health institutions were included in our study, so it might not accurately represent the entire country. To our knowledge, however, this is the first study to investigate knowledge of toxoplasmosis knowledge of HCWs in Zambia. This will inform possible future research into this critcal information gap in the management of toxoplasmosis.

## Conclusion

Our findings revealed that clinicians had the highest levels of knowledge about the disease, while nurses had the highest levels of adherence to good practices. Overall, our study showed low knowledge of toxoplasmosis and poor adherence to toxoplasma preventive practices in HCWs at Namwala and Ndola Teaching Hospital. There is need to institute continuous medical education using a complementary approach targeting knowledge in nurses and practices in clinicians.

### Recommendations and future direction

In our study, we found that staff with less experience had more knowledge and nurses had better practices despite lower levels of knowledge. These findings suggest that there is a need to improve continuing medical education programs for healthcare staff in order to increase their knowledge and adherence to good practices related to toxoplasmosis. Additionally, there may be a need to investigate why certain staff groups have better practices despite lower levels of knowledge, in order to understand how to improve overall knowledge and practice in the healthcare setting. We propose more studies to understand the role of medical education in toxoplasma prevention and control in the Zambian context.

## Supporting information

S1 File Study questionnaire. (DOC)

S2 File STROBE checklist. (DOCX)

S3 File PLOS ONE clinical studies checklist. (DOCX)

S4 File Study dataset. (CSV)

S5 File Study ethics approval. (PDF)

S6 File PLOS questionnaire. (DOCX)

## References

[1] Chandrasena N, Herath R, Rupasinghe N, Samarasinghe B, Samaranayake H, Kastuririratne A, et al. Toxoplasmosis awareness, seroprevalence and risk behavior among pregnant women in the Gampaha district, Sri Lanka. Pathog Glob Health. 2016 Feb 17;110(2):62–7. doi: 10.1080/20477724.2016.1173325 27092763 PMC4894262

[2] Omonijo AO, Kalinda C, Mukaratirwa S. Toxoplasma gondii Infections in Animals and Humans in Southern Africa: A Systematic Review and Meta-Analysis. Pathogens. 2022 Feb 1;11(2). doi: 10.3390/pathogens11020183 35215126 PMC8880191

[3] Hammond-Aryee K, Esser M, Van Helden P. Toxoplasmosis in South Africa-Old Disease in A New Context. Vol. 4, Journal of Natural Sciences Research www.iiste.org ISSN. Online; 2014.

[4] Alvarado-Esquivel C, Sánchez-Anguiano LF, Berumen-Segovia LO, Hernández-Tinoco J, Rico-Almochantaf YDR, Cisneros-Camacho A, et al. Knowledge and practices of toxoplasmosis among clinical laboratory professionals: A cross-sectional study in Durango, Mexico. Int J Environ Res Public Health. 2017 Nov 18;14(11). doi: 10.3390/ijerph14111413 29156547 PMC5708052

[5] Flegr J, Prandota J, Sovičková M, Israili ZH. Toxoplasmosis—A global threat. Correlation of latent toxoplasmosis with specific disease burden in a set of 88 countries. PLoS One. 2014 Mar 24;9(3). 10.1371/journal.pone.0090203 PMC3963851 24662942

[6] Elsheikha HM, Marra CM, Zhu XQ. Epidemiology, pathophysiology, diagnosis, and management of cerebral toxoplasmosis. Clin Microbiol Rev. 2020 Nov 25;34(1):1–28. doi: 10.1128/CMR.00115-19 33239310 PMC7690944

[7] Laboudi M, Ait Hamou S, Mansour I, Hilmi I, Sadak A. The first report of the evaluation of the knowledge regarding toxoplasmosis among health professionals in public health centers in Rabat, Morocco. Trop Med Health. 2020 Apr 9;48(1):17. doi: 10.1186/s41182-020-00208-9 32292287 PMC7144052

[8] Robert-Gangneux F, Dion S. Toxoplasmosis and pregnancy. Canadian Family Physician. 2014 Oct 1;60(4):334. 24733322 PMC4046541

[9] Kota AS, Shabbir N. Congenital Toxoplasmosis. StatPearls. 2022 Jun 27; 31424812

[10] McAuley JB. Congenital toxoplasmosis. J Pediatric Infect Dis Soc. 2014;3(SUPPL1):S30. doi: 10.1093/jpids/piu077 25232475 PMC4164182

[11] Efunshile AM, Elikwu CJ, Jokelainen P. Toxoplasmosis–awareness and knowledge among medical doctors in Nigeria. PLoS One. 2017 Dec 1;12(12). doi: 10.1371/journal.pone.0189709 29261738 PMC5736225

[12] Zumla A, Savva D, Hira SK, Luo NP, Kaleebu P, Sempala SK, et al. Toxoplasma serology in Zambian and Ugandan patients infected with the human immunodeficiency virus. Trans R Soc Trop Med Hyg. 1991 Jan 1;85(2):227–9. doi: 10.1016/0035-9203(91)90034-v 1887478

[13] Daka V. Seroprevalence and Risk Factors of Toxoplasmosis in individuals attending Chipokotamayamba Clinic in Ndola, Zambia. Lusaka, Zambia; 2015.

[14] Frimpong C, Makasa M, Sitali L, Michelo C. Seroprevalence and determinants of toxoplasmosis in pregnant women attending antenatal clinic at the university teaching hospital, Lusaka, Zambia. BMC Infect Dis [Internet]. 2017 Jan 5 [cited 2021 Feb 23];17(1):10. Available from: http://bmcinfectdis.biomedcentral.com/articles/10.1186/s12879-016-2133-7 28056829 10.1186/s12879-016-2133-7 PMC5216584

[15] Pawlowski ZS, Gromadecka-Sutkiewicz M, Skommer J, Paul M, Rokossowski H, Suchocka E, et al. Impact of health education on knowledge and prevention behavior for congenital toxoplasmosis: The experience in Poznań, Poland. Health Educ Res. 2001;16(4):493–502. 11525395 10.1093/her/16.4.493

[16] Dabritz HA, Conrad PA. Evaluation of an educational handout on knowledge about toxoplasmosis. Sci Med (Porto Alegre). 2010 Feb 22;20(1):51.

[17] Contiero-Toninato AP, Cavalli HO, Marchioro AA, Ferreira ÉC, Caniatti MC da CL, Breganó RM, et al. Toxoplasmosis: An examination of knowledge among health professionals and pregnant women in a municipality of the State of Paraná. Rev Soc Bras Med Trop. 2014;47(2):198–203. 24861294 10.1590/0037-8682-0016-2014

[18] Da Silva LB, De Oliveira RDVC, Da Silva MP, Bueno WF, Amendoeira MRR, Neves EDS. Knowledge of toxoplasmosis among doctors and nurses who provide prenatal care in an endemic region. Infect Dis Obstet Gynecol. 2011;2011. 10.1155/2011/750484 PMC3124125 21747644

[19] Maqsood T, Shahzad K, Naz S, Simsek S, Afzal MS, Ali S, et al. A Cross-Sectional Study on the Association Between Risk Factors of Toxoplasmosis and One Health Knowledge in Pakistan. Front Vet Sci [Internet]. 2021 Nov 18 [cited 2023 May 26];8:751130. Available from: /pmc/articles/PMC8637412/ doi: 10.3389/fvets.2021.751130 34869724 PMC8637412

[20] Aguirre AA, Longcore T, Barbieri M, Dabritz H, Hill D, Klein PN, et al. The One Health Approach to Toxoplasmosis: Epidemiology, Control, and Prevention Strategies. Ecohealth [Internet]. 2019 Jun 15 [cited 2023 Jun 26];16(2):378. Available from: /pmc/articles/PMC6682582/ doi: 10.1007/s10393-019-01405-7 30945159 PMC6682582

[21] Malama S, Johansen TB, Muma JB, Munyeme M, Mbulo G, Muwonge A, et al. Characterization of Mycobacterium bovis from humans and cattle in Namwala District, Zambia. Vet Med Int. 2014;2014. 10.1155/2014/187842 PMC4009325 24847441

[22] Monde N, Munyeme M, Muwonge A, Muma JB, Malama S. Characterization of non-tuberculous mycobacterium from humans and water in an Agropastoral area in Zambia. BMC Infect Dis. 2018 Jan 8;18(1):1–7. 29310592 10.1186/s12879-017-2939-y PMC5759224

[23] Chanda W, Manyepa M, Chikwanda E, Daka V, Chileshe J, Tembo M, et al. Evaluation of antibiotic susceptibility patterns of pathogens isolated from routine laboratory specimens at Ndola Teaching Hospital: A retrospective study. Abuelo A, editor. PLoS One. 2019 Dec 23;14(12):e0226676. doi: 10.1371/journal.pone.0226676 31869354 PMC6927611

[24] Mwansa NJ, Daka V, Mulenga D, Mfune RL, Mukanga B, Nyirenda C, et al. Correlates of Seizure Control Among Patients with Epilepsy at Two Referral Hospitals in Zambia. International Journal of Translational Medical Research and Public Health. 2020 Sep 9;4(2):130–8.

[25] Dean AG, Sullivan KM, Soe MM. OpenEpi: Open Source Epidemiologic Statistics for Public Health [Internet]. 2013 [cited 2022 Dec 23]. http://www.openepi.com/Menu/OE_Menu.htm

[26] Harris PA, Taylor R, Thielke R, Payne J, Gonzalez N, Conde JG. Research electronic data capture (REDCap)—a metadata-driven methodology and workflow process for providing translational research informatics support. J Biomed Inform. 2009 Apr;42(2):377–81. doi: 10.1016/j.jbi.2008.08.010 18929686 PMC2700030

[27] He Y. Missing data analysis using multiple imputation: getting to the heart of the matter. Circ Cardiovasc Qual Outcomes. 2010 Jan;3(1):98–105. doi: 10.1161/CIRCOUTCOMES.109.875658 20123676 PMC2818781

[28] Azlan AA, Hamzah MR, Sern TJ, Ayub SH, Mohamad E. Public knowledge, attitudes and practices towards COVID-19: A cross-sectional study in Malaysia. Tu WJ, editor. PLoS One. 2020 May 21;15(5):e0233668. 32437434 10.1371/journal.pone.0233668 PMC7241824

[29] Wang L, Abualfoul M, Oduor H, Acharya P, Cui M, Murray A, et al. A cross-sectional study of knowledge, attitude, and practice toward COVID-19 in solid organ transplant recipients at a transplant center in the United States. Front Public Health. 2022 Sep 23;10:3135. 10.3389/fpubh.2022.880774 PMC9539443 36211649

[30] Hadush Desta A. Knowledge, Attitude and Practice of Community Towards Zoonotic Importance of *Toxoplasma* Infection in Central Afar Region, North East Ethiopia. International Journal of Biomedical Science and Engineering. 2015 Dec 3;3(6):74.

[31] Onduru OG, Rumisha SF, Munyeme M, Phiri AM. Evaluation of the level of awareness of congenital toxoplasmosis and associated practices among pregnant women and health workers in tanzania’s temeke district in dar es Salaam. Afr Health Sci. 2019 Dec 1;19(4):3027–37. doi: 10.4314/ahs.v19i4.24 32127878 PMC7040330

[32] Mahfouz M, Elmahdy M, Bahri A, Mobarki Y, Altalhi A, Barkat N, et al. Knowledge and attitude regarding toxoplasmosis among Jazan University female students. Saudi J Med Med Sci. 2019;7(1):28. doi: 10.4103/sjmms.sjmms_33_17 30787854 PMC6381842

[33] Nwagu EN, Abugu LI, Yohanna W, Eze DN, Ononuju AH, Obayi AN. Behaviour change communication for control of tuberculosis by healthcare workers in DOTS facilities in Nigeria. PAMJ 2020; 36:306. 2020 Aug 19;36(306):1–10. doi: 10.11604/pamj.2020.36.306.21640 33282089 PMC7687482

[34] Khudair FW. Knowledge of Prenatal Care Nurses toward Management of Toxoplasmosis in Pregnant Women. Iraqi National Journal of Nursing Specialties [Internet]. 2013 Jun 30 [cited 2023 Jun 26];26(1):58–62. Available from: https://injns.uobaghdad.edu.iq/index.php/INJNS/article/view/162

