# Supplementary material for: Knowledge and practices of toxoplasmosis among healthcare workers at two large referral hospitals in Zambia: Implications on the One Health Approach
Source: PLOS Glob Public Health. 2023 Aug 15;3(8):e0002235. doi: 10.1371/journal.pgph.0002235 (PMC10426967; doi:10.1371/journal.pgph.0002235)
Supplement: S1 File — (DOC) [file pgph.0002235.s001.doc]

**KAP for Healthcare workers**

*Toxo among Healthcare workers*

*Page 1*

| A1. | Record ID | __________________________________ |
| --- | --- | --- |
|  |  |
|  |  |  |
| A2. | District | Namwala |
|  |  | Ndola |
|  |  | Nyimba |
|  |  |  |
| A3. | Health Facility | __________________________________ |
|  |  |
|  |  |  |
| A4. | Section of work | __________________________________ |
|  |  |


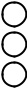


**Demographic and Background Characteristics of participants**

| B1. | Gender | Male |
| --- | --- | --- |
|  |  | Female |
|  |  |  |
| B2. | Age | __________________________________ |
|  |  |
|  |  |  |
| B3. | Residence withing the district | __________________________________ |
|  |  |
|  |  |  |
| B4. | Marital Status | Single |
|  |  | Married/Cohabiting |
|  |  | Divorced/Separation |
|  |  | Widowed |
|  |  |  |
| B5. | Designation as a healthworker | Nurse |
|  |  | Doctor |
|  |  | Clinical Officer/medical licentiate |
|  |  | Laboratory staff |
|  |  | Pharmacy staff |
|  |  | Other |
|  |  |  |
| B6. | Highest Education attained | Primary education |
|  |  | Secondary education |
|  |  | College Education |
|  |  | University education |
|  |  |  |
| B7. | How many years of experience as a healthworker do | __________________________________ |
| you have | |
|  |  |  |
| B8. | Do you know when and how to treat toxoplasmosis? | Yes |
|  |  | No |
|  |  | I don't know |
|  |  |  |
| B9. | Have you ever tested for toxoplasmosis | Yes |
|  |  | No |
|  | |  |
| B10. Have you ever screened a patient/ client for | | Yes |
| toxoplasmosis | | No |


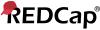

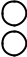

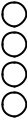

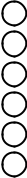

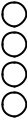

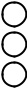

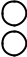

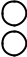


[projectredcap.org](https://projectredcap.org/)

*Page 2*

| B11. | Have you ever seen a clinical case (clinicians) | Yes |
| --- | --- | --- |
| or handled a sample of toxoplasmosis (for lab staff)? | | No |
|  |  |  |
| B12. | Do you work in the ART section or MCH sections of | Yes |
| the facility | | No |


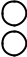

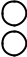


**Questions on Knowledge**

| C1. | Toxoplasma gondii is a/an | bacterium |
| --- | --- | --- |
|  |  | virus |
|  |  | parasite |
|  |  | fungi |
|  |  | insect |
|  |  |  |
| C2. | Toxoplasma gondii can infect humans | Yes |
|  |  | No |
|  |  | I don't know |
|  |  |  |
| C3. | Toxoplasmosis is caused by a toxin | Yes |
|  |  | No |
|  |  | I don't know |
|  |  |  |
| C4. | Can toxoplasmosis be treated | Yes |
|  |  | No |
|  |  | I don't know |
|  |  |  |
| C5. | Which of the following medicine can be used for | Pyrimethamine |
| management of toxoplasmosis | | Sulfadiazine |
|  |  | Sulfadoxine |
|  |  | Folic acid |
|  |  | Spiramycine |
|  |  | I do not know |
|  |  |  |
| C6. | Can pregnant women develop complications due to | Yes |
| toxoplasma infection? | | No |
|  |  | I don't know |
|  |  |  |
| C7. | Can unborn and new/born children develop serious | Yes |
| complication after infection with toxoplasmosis | | No |
|  |  | I don't know |
|  |  |  |
| C8. | Can a baby with toxoplasmosis have vision and | Yes |
| mental problems | | No |
|  |  | I don't know |
|  |  |  |
| C9. | Chorioretinitis is a clinical manifestation of | Yes |
| toxoplasmosis | | No |
|  |  | I don't know |
|  | |  |
| C10. Hydrocephalus in children is a clinical | | Yes |
| manifestation of toxoplasmosis | | No |
|  |  | I don't know |
|  | |  |
| C11. Encephalitis is a clinical manifestation of | | Yes |
| toxoplasmosis | | No |
|  |  | I don't know |


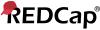

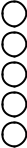

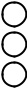

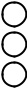

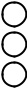

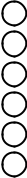

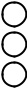

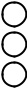

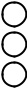

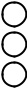

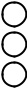

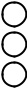


[projectredcap.org](https://projectredcap.org/)

*Page 3*

| C12. | Mental retardation is a clinical manifestation of | Yes |
| --- | --- | --- |
| toxoplasmosis | | No |
|  |  | I don't know |
|  |  |  |
| C13. | Disorientation is a clinical manifestation of | Yes |
| toxoplasmosis | | No |
|  |  | I don't know |
|  |  |  |
| C14. | Intracranial calcification is a clinical | Yes |
| manifestation of toxoplasmosis | | No |
|  |  | I don't know |
|  |  |  |
| C15. | Toxoplasma can infect pigs | Yes |
|  |  | No |
|  |  | I don't know |
|  |  |  |
| C16. | Toxoplasma can infect sheep | Yes |
|  |  | No |
|  |  | I don't know |
|  |  |  |
| C17. | Toxoplasma can infect chickens | Yes |
|  |  | No |
|  |  | I don't know |
|  |  |  |
| C18. | Toxoplasma can infect dogs | Yes |
|  |  | No |
|  |  | I don't know |
|  |  |  |
| C19. | Toxoplasma can infect cats | Yes |
|  |  | No |
|  |  | I don't know |
|  |  |  |
| C20. | Toxoplasma can infect mosquitoes | Yes |
|  |  | No |
|  |  | I don't know |
|  |  |  |
| C21. | Toxoplasma gondii can be shed in faeces of humans | Yes |
|  |  | No |
|  |  | I don't know |
|  |  |  |
| C22. | Toxoplasma gondii can be shed in faeces of fish | Yes |
|  |  | No |
|  |  | I don't know |
|  |  |  |
| C23. | Toxoplasma gondii can be shed in faeces of | Yes |
| chicken | | No |
|  |  | I don't know |
|  |  |  |
| C24. | Toxoplasma gondii can be shed in faeces of cats | Yes |
|  |  | No |
|  |  | I don't know |
|  |  |  |
| C25. | Toxoplasma gondii can be shed in faeces of cattle | Yes |
|  |  | No |
|  |  | I don't know |


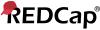

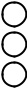

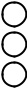

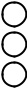

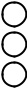

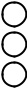

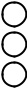

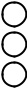

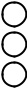

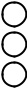

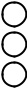

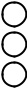

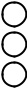

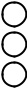

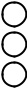


[projectredcap.org](https://projectredcap.org/)

*Page 4*

| C26. | Toxoplasma gondii infection can be meatborne | Yes |
| --- | --- | --- |
|  |  | No |
|  |  | I don't know |
|  |  |  |
| C27. | Toxoplasma gondii infection can be waterborne | Yes |
|  |  | No |
|  |  | I don't know |
|  |  |  |
| C28. | Clinical toxoplasmosis may involve the central | Yes |
| nervous system | | No |
|  |  | I don't know |
|  |  |  |
| C30. | Clinical toxoplasmosis may affect blood cells | Yes |
|  |  | No |
|  |  | I don't know |


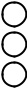

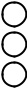

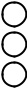

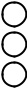


**Questions on risk factors**

| D1. | Do you have domestic cat(s) at home | Yes |
| --- | --- | --- |
|  |  | No |
|  |  |  |
| D2. | If yes to D1, Do you change cat litter/clean cat | Yes |
| premises | | No |
|  |  |  |
| D3. | Have you ever been involved in farming or | Yes |
| gardening activities? | | No |
|  |  |  |
| D4. | If yes to D3, do you wear gloves when farming or | Yes |
| gardening | | No |
|  |  |  |
| D5. | Do you thoroughly wash and peel fruits and | Yes |
| vegetables before eating? | | No |
|  |  |  |
| D6. | Have you ever eaten raw/ undercooked or cured | Yes |
| meat? | | No |
|  |  |  |
| D7. | Have you ever eaten raw fruits/ vegetables? | Yes |
|  |  | No |
|  |  |  |
| D8. | Do you drink raw milk? | Yes |
|  |  | No |
|  |  |  |
| D9. | Do you drink untreated water? | Yes |
|  |  | No |


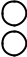

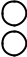

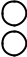

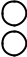

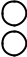

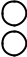

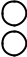

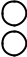

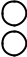


GPS- latitude

__________________________________

GPS- longitude

__________________________________


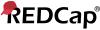


[projectredcap.org](https://projectredcap.org/)
