# Supplementary material for: Knowledge and practices of toxoplasmosis among healthcare workers at two large referral hospitals in Zambia: Implications on the One Health Approach
Source: PLOS Glob Public Health. 2023 Aug 15;3(8):e0002235. doi: 10.1371/journal.pgph.0002235 (PMC10426967; doi:10.1371/journal.pgph.0002235)
Supplement: S5 File — (PDF) [file pgph.0002235.s005.pdf]

TROPICAL DISEASES **RESEARCH CENTRE**
Tel/Fax +260212 615444
P O Box 71769
tdrc-ethics@tdrc.org.zm
NDOLA, ZAMBIA

TDRC RESEARCH ETHICS COMMITTEE
IRB REGISTRATION NUMBER : 00002911
FWA NUMBER : 00003729

TRC/C4/08/2021

21st August, 2021

TROPICAL DISEASES RESEARCH CENTRE
ETHICS REVIEW COMMITTEE
21 AUG 2021
P.O. BOX 71769
NDOLA, ZAMBIA

Mr Victor Daka
Principal Investigator
University of Zambia
School of Veterinary Medicine
LUSAKA

Dear Mr Daka,

RE: ETHICAL APPROVAL OF STUDY PROTOCOL

Reference is made to your letter dated 30th July 2021 in which you were applying for ethical clearance for the protocol entitled **"Characterization of Toxoplasmosis in Selected District in Zambia."**

On behalf of the Chairman of the TDRC Research Ethics Committee (REC), I am pleased to inform you that your protocol and other supporting documents were reviewed and granted ethical approval based on the following conditions:

Since your study is cross-sectional, you will require to obtain consent from your study participants. Ensure that you obtain authorization from respective District Health Directors in the selected districts you would be conducting your research prior to commencement of research. Furthermore, ensure that your Data Collection tools and consent forms are translated into local language as some of the participants may not understand the English language.

Should there be any protocol modifications, amendments or violations, you are required to notify the REC and submit the protocol amendments for approval.

You are now required to submit your protocol to the NHRA for final approval following the link: **https://www.nhra.org.zm**. A final report to the study should be submitted to the REC Secretariat at the end of the study.

This approval is valid for the period, **21st August, 2021 to 20th August 2022.**

The Committee wishes you success in academic work and execution of the study.

Yours faithfully,
TROPICAL DISEASES RESEARCH CENTRE

Sydney Mwanza
DEPUTY SECRETARY – TDRC REC

CC-TDRC REC Chairperson
